# Clinical utility of liquid biopsy in bladder cancer: The beginning of a new era

**DOI:** 10.1016/j.jlb.2024.100271

**Published:** 2024-10-18

**Authors:** Eric Jia, Gautum Agarwal

**Affiliations:** a Department of Computer Science, Washington University in St. Louis, St. Louis, MO, USA; b Mercy Hospital, St. Louis, MO, USA

**Keywords:** Bladder cancer, Precision medicine, Bladder cancer management, Urine-based molecular diagnostics, Urothelial carcinoma, Urine diagnostics, Quality of life, Review

## Abstract

Bladder cancer (BC) is the sixth most prevalent cancer in the U.S. and the most expensive to treat. Integrating precision medicine into BC management promises improved outcomes and reduced costs. This review explores current treatment paradigms and the transformative potential of urine-based molecular diagnostics. Treatments for BC range from transurethral resection and intravesical therapy for non-muscle invasive bladder cancer (NMIBC) to neoadjuvant chemotherapy and radical cystectomy for muscle-invasive bladder cancer (MIBC). Recent breakthroughs include enfortumab vedotin with pembrolizumab for advanced urothelial carcinoma, PD-1 immunotherapy for minimal residual disease (MRD)-positive patients and erdafitinib for NMIBC. Traditional diagnostic methods like cystoscopy, urine cytology, and imaging have limitations; urine-based diagnostics, particularly urinary tumor DNA (utDNA) analysis, offer a non-invasive, sensitive, and cost-effective alternative. These diagnostics facilitate personalized treatment, monitor therapy response, detect MRD, and enable earlier cancer detection. Incorporating urine-based diagnostics into clinical practice can reduce healthcare costs and improve patient quality of life. This review highlights the need for these diagnostics in routine BC management and emphasizes the impact of recent therapeutic advances.

## 1 Introduction

Bladder cancer (BC) is the sixth most prevalent cancer in the U.S., with over 83,000 new patients reported annually [1]. However, BC has the highest lifetime treatment costs of all cancers, ranging from $89,287 to $202,203 per patient, depending on the country [2]. In 2021, the cost of treating BC in the United States was estimated to exceed $6.5 billion [1]. By 2030, Medicare is projected to pay $11.6 billion in costs related to BC [3].

The advancement of precision medicine, which aims to personalize medical treatment using individual-level characteristics, holds strong promise to improve therapeutic efficacy and minimize adverse effects, as evidenced by recent breakthroughs in bladder cancer management.

This review examines the current treatment paradigm of bladder cancer. It highlights the cost-saving and transformative potential of urine-based molecular diagnostics in revolutionizing the bladder cancer care continuum, including treatment selection, monitoring therapy response, detecting minimal residual disease (MRD), and eventually early cancer detection.

## 2 Current treatment paradigm and new treatment breakthrough for bladder cancer

Bladder cancer treatment is highly complex, often requiring a multi-modal approach contingent upon both the tumor's stage and grade. Among newly diagnosed bladder cancer patients, non-muscle invasive bladder cancer (NMIBC) constitutes approximately 70 % of cases, while muscle invasive bladder cancer (MIBC) accounts for the remaining 30 % [4].

NMIBC is typically managed through transurethral resection (TUR), a surgical procedure where the tumor is removed through the urethra, followed by intravesical therapy, where medication is delivered directly into the bladder to kill any remaining cancer cells [5]. Common intravesical therapies include chemotherapy or Bacillus Calmette-Guerin (BCG), an immunotherapy that stimulates the immune system to fight cancer cells. However, up to 40 % of NMIBC patients do not respond to BCG, leading to disease recurrence and progression [6]. For these patients, a radical cystectomy involving the removal of the bladder emerges as the primary treatment option.

In contrast, MIBC frequently necessitates more aggressive interventions. The current standard of care for patients with localized MIBC involves neoadjuvant cisplatin-based chemotherapy followed by radical cystectomy [7]. Even with chemotherapy and surgery, the recurrence of cancer is common as evidenced by a 5-year survival rate of 50–60 % [7].

The EV-302 study evaluated the efficacy of enfortumab vedotin (EV), a novel drug in a newly developed class of treatments termed antibody-drug conjugates (ADCs), plus pembrolizumab versus standard chemotherapy in untreated advanced urothelial carcinoma (UC) [8]. Powles et al. reported that the combination therapy resulted in a median overall survival (OS) benefit of 31.5 months compared to 16.1 months for chemotherapy, breaking the 20-year record where platinum-based chemotherapy has been the standard of care for first-line treatment in previously untreated locally advanced metastatic urothelial carcinoma [8]. This study was featured in Nature's Top 10 Breakthrough List in 2023 for its role in EV-302 and the progress of ADCs in BC [9].

PD-1 immunotherapy treatment has achieved another breakthrough for advanced bladder cancer, for which there have been no major treatment advances in the past 30 years. In the IMvigor010 trial, Powles et al. reported that atezolizumab improved disease-free survival (DFS) and OS in patients who were MRD ctDNA positive, as measured by a tissue-informed blood-based MRD assay [10]. A positive surveillance analysis has been announced from the prospective phase III IMvigor011 trial in MIBC at the EAU Congress 2024 [11].

## 3 Current diagnostic tools in clinical practice

The array of diagnostic tools available for bladder cancer detection each comes with its own advantages and disadvantages. Cystoscopy involves the insertion of a cystoscope, a thin tube with a camera and light, into the bladder through the urethra to observe the bladder lining. This allows for direct visualization of the urethra and bladder to observe these structures for the presence of disease. Additionally, when using the cystoscope, a biopsy can be performed by inserting an additional object through the cystoscope to collect a tissue sample in the bladder which can be examined further for signs of cancer [5]. While cystoscopy is the gold standard for diagnosis, it is invasive and can be uncomfortable for patients. It may also miss flat lesions or tumors located in areas that are not easily visible [12].

Urine cytology, on the other hand, is a non-invasive test that analyzes a urine sample for the presence of cancer cells shed from the lining of the bladder. Although convenient and easy to perform, current urine cytology tests have limited sensitivity, particularly for low-grade tumors, and can yield false-negative results [12].

Imaging tests like computed tomography (CT) scans or urograms can provide detailed images of the bladder and surrounding tissues to help assess the extent of tumor invasion and whether the cancer has metastasized further into the urinary tract. However, these tests are expensive, potentially expose patients to radiation (for CT scans), and may not detect smaller tumors in the bladder [12].

## 4 The emerging value of urine molecular diagnostics

The dawn of urine-based molecular diagnostics, particularly the analysis of urinary tumor DNA (utDNA), has ushered in a new era of precision medicine for bladder cancer. This non-invasive approach involves analyzing DNA fragments shed from tumor cells in the urine, offering significant information to aid in patient care. Zhang and colleagues reported that urine-based molecular diagnostics provide a valid and complete genetic profile of bladder cancer and represent an accurate proxy for genotyping and monitoring newly diagnosed BC [13]. Using matched tissue-blood-urine-peripheral blood mononuclear cell (PBMC), the authors demonstrated highly concordant mutational landscapes captured by tumor tissue DNA (tDNA) or utDNA, but not by blood circulating tumor DNA (ctDNA) [13]. It was noted that higher preoperative utDNA or tDNA abundance correlated with worse relapse-free survival [13]. Potentially actionable genomic alterations including fibroblast growth factor receptor 2/3 (*FGFR2/3*), receptor tyrosine-protein kinase erbB-2 (*ERBB2)*, and phosphatidylinositol-4,5-bisphosphate 3-kinase catalytic subunit alpha (*PIK3CA*) were identified in utDNA [13].

Furthermore, Zhang and colleagues demonstrated the value of utDNA MRD analysis in the neoadjuvant setting for MIBC compared to traditional radiographic assessments and conventional biomarkers in predicting pathological outcomes following immune checkpoint blockade with toripalimab, a PD-1 immunotherapy drug [14]. This study highlighted the potential of utDNA MRD testing to guide bladder-sparing treatment decision-making, identifying patients who could avoid or postpone radical cystectomy.

Rose and colleagues reported that utDNA and ctDNA effectively detect MRD to guide personalized treatment decisions and improve patient outcomes. Their study revealed that utDNA could accurately reflect the tumor's genomic landscape, facilitating targeted therapy and monitoring treatment response with high sensitivity and specificity [15]. Frequent monitoring of BC through urine diagnostics offers a less invasive alternative to traditional methods like cystoscopy, significantly reducing patient discomfort and healthcare costs. This approach aligns with the evidence presented, which supports the integration of utDNA analysis into routine clinical practice for enhanced patient care and cost-efficiency in managing bladder cancer.

Most recently, urine has been actively used in clinical trials for patient selection as evidenced in the TAR-210 study which may shift the treatment landscape of NMIBC. At the ASCO-GU24 conference, Li et al. reported the use of urine-based testing for patient selection and genomic characterization in patients with *FGFR* alteration-positive NMIBC treated with TAR-210, an intravesical drug delivery system, designed to continuously release erdafitinib directly into the bladder [16]. At the recent AUA24 conference, Vilaseca et al. reported the result of an open-label, multicenter, multi-cohort Phase 1 study of the safety and efficacy of TAR-210, reporting a 90 % recurrence-free survival (RFS) and 90 % complete response (CR) in patients with high-risk and intermediate-risk NMIBC with select FGFR alterations, respectively [17]. At the same AUA24 conference, Li announced the phase 3 MoonRISe-1study to evaluate TAR-210 in intermediate-risk NMIBC patients with *FGFR* alterations [18]. In both cases, urine or tissue-based testing is used to select patients in these studies.

## 5 Early cancer detection using urine: A paradigm shift

The non-invasive nature and increasing sensitivity and specificity of urine tests render them invaluable tools for the early cancer detection of bladder cancer. Urinary molecular pathology has demonstrated its potential to detect urothelial bladder cancer at its earliest stages, providing a crucial window for curative intervention. Early detection significantly improves survival rates, emphasizing the importance of integrating urine-based diagnostics into routine clinical practice.

## 6 Economic implications and quality of life using urine-based molecular diagnostics

Bladder cancer management is expensive due to high recurrence rates and the need for continuous surveillance. Integrating urine-based molecular diagnostics may substantially reduce healthcare operational costs, providing a valuable tool to facilitate physician's clinical decision-making while alleviating the financial burden for patients and the healthcare system.

Liquid biopsy-based molecular diagnostics streamline treatment selection, minimize invasive procedures such as cystoscopy, and facilitate earlier detection to enable earlier intervention. For example, a urine-based test determining a patient's eligibility for targeted therapy can circumvent the large expenses of potentially ineffective treatments, eradicating tumors while saving significant financial and medical resources. Detection of minimal residual disease through urine-based tests may enable organ preservation and improve the quality of life for patients with MIBC.

For instance, Clark et al. reported that the total cost of bladder cancer care in 2021 was estimated to exceed $6.5 billion, based on an estimated 83,532 newly diagnosed BC patients of all stages in the United States during that year, with a projected total cost of treatment of $2,584,783,728 [1]. The average cost per patient varied from $19,521 (stage 0a) to $169,533 (metastatic disease) [1]. For the 75,760 patients expected to have a recurrence in 2021, an additional cost of $3,953,096,316 was estimated at an average cost per patient of $52,179 [1]. The recent advancements in urine-based patient selection for TAR-210 therapy and urine-informed MRD study for bladder preservation may offer hope for improved quality of life while potentially saving hundreds of millions of dollars at the societal level.

## 7 Conclusion

Incorporating urine-based diagnostics into clinical practice represents a significant leap forward in bladder cancer management. These non-invasive, sensitive, and cost-effective tools have the potential to revolutionize precision medicine, enabling personalized treatment strategies that optimize patient outcomes while reducing healthcare expenditures. As novel research continues to unravel the intricacies of urinary molecular markers, we anticipate an even greater impact of urine-based diagnostics on the future of bladder cancer care to detect and treat bladder cancer early. A new era of bladder cancer diagnostics and treatment has begun.

## Ethical approval patient consent

No ethical approvals or patient consent were necessary for the study.

## Declaration of competing interest

The authors declare that they have no known competing financial interests or personal relationships that could have appeared to influence the work reported in this paper.
